# Reviewing the definition of crisis in dementia care

**DOI:** 10.1186/1471-2318-13-10

**Published:** 2013-02-01

**Authors:** Janet MacNeil Vroomen, Judith E Bosmans, Hein PJ van Hout, Sophia E de Rooij

**Affiliations:** 1 Department of Internal Medicine, Section of Geriatric Medicine, Academic Medical Center, University of Amsterdam, Amsterdam, The Netherlands; 2 Department of Health Sciences and EMGO Institute for Health and Care Research, Faculty of Earth and Life Sciences, VU University Amsterdam, Amsterdam, The Netherlands; 3 Department of General Practice, EMGO-Institute, VU University Medical Centre, Amsterdam, The Netherlands; 4 Academic Medical Center, Ouderengeneeskunde/KOZ, Meibergdreef 9, F4-218, Amsterdam, 1105 AZ, Netherlands

**Keywords:** Crisis, Dementia care, Definition, Operational framework

## Abstract

**Background:**

Crisis is a term frequently used in dementia care lacking a standardized definition. This article systematically reviews existing definitions of crisis in dementia care literature to create a standardized definition that can be utilized for research, policy and clinical practice.

**Methods:**

We systematically searched for articles containing definitions of crisis in the context of dementia care. We created an operational framework of crisis based on retrieved definitions. Recommendations to address crisis situations were reviewed and classified according to care settings.

**Results:**

Abstracts and titles of 1,113 articles, screened from PubMed and EMBASE, were narrowed down to 27 articles. After review, crisis in dementia was defined as *a process where a stressor causes an imbalance requiring an immediate decision to be made which leads to a desired outcome and therefore a resolution of the crisis*. *If the crisis is not resolved, the cycle continues.* Recommendations for resolving crisis involving persons with dementia and their caregivers include awareness therapy after diagnosis and increased contact with general practitioners, case manager consultations, caregiver support and education. Furthermore, nursing home staff should be attuned to the environmental, physical and psychological needs of persons with dementia.

**Conclusions:**

This is the first article to review the definition of crisis in the context of dementia care. A review of the literature indicated that the definition of a crisis is idiosyncratic. Therefore, it is difficult to prevent or plan for all crises. We used an operational framework to compile types of crisis stressors and recommendations from the crisis literature based on three different perspectives; the person with the dementia, the caregiver and the healthcare providers.

## Background

In medical Latin, the word *crisis* denotes the turning point of a disease and was originally derived from the Greek word *krinein,* meaning ‘to decide’ [1]. The general sense that crisis is a ‘decisive point’ dates from the early 17th century [1]. The Oxford dictionary defines crisis as “a time of intense difficulty or danger (…) a time when a difficult or important decision must be made … the turning point of a disease when an important change takes place, indicating either recovery or death” [2]. However, in the context of dementia care, literature, health policy and care practice, crisis is mentioned most often in the context of difficulty or danger, and not solely as a turning point of the disease alone [3-5].

Various definitions of the word crisis are used in the medical literature. These definitions include the perspectives of the person with dementia, their caregiver(s) and health care providers. A standardized definition of crisis is needed in order to systematically evaluate the impact of interventions on the occurrence of crisis situations. Therefore, the objective of this article was to systematically review the dementia literature to identify crisis definitions and to propose a standardized definition for clinical practice and future research.

First, we provided definitions of crisis pertaining to dementia care from the literature. Second, we created an operational framework based on key elements from all definitions identified. Third, we reviewed and summarized the literature’s recommendations to address crisis.

## Methods

### Search strategy

We followed the PRISMA guidelines to develop our search strategy [6]. PubMed (1946 to March 2011) and Embase (1947 to March 2011) search engines were explored with a librarian. Mesh terms included:

• Dementia

• Crisis intervention

• Emergencies

• Human.

Additionally, the references from the articles were reviewed.

### Inclusion criteria strategy

Two reviewers (JB and JM-V) independently selected studies to include in the systematic review by screening titles and abstracts of all publications downloaded from the electronic databases. We used the following five inclusion criteria:

1. Articles written in English

2. Participants 65 years or older

3. Studies in humans

4. Dementia related crisis, crisis intervention or emergency themes related to dementia care

5. Crisis is described or defined.

During a consensus meeting between the two reviewers, opinions regarding potentially relevant studies were discussed and selection was finalized. The final decision on the inclusion of a study was based on the full article.

### Data extraction and analysis

The data that was compiled from all studies included:

• information on the type of study (e.g. randomized controlled trial)

• the crisis definition used

• the perspective (person with dementia, informal caregiver, heath care provider)

• the recommendations pertaining to the crisis.

The definitions and explanations of crisis were then organized into tables, and systematically compared. Frequently occurring concepts included in these definitions and explanations of crisis were synthesized and included in the definition and operational framework. We examined how these concepts fit together to summarize crisis and we put these concepts into an operational framework. We identified stressors, types of crisis and solutions from various perspectives based on our literature search. From this information, we created the definition. The operational framework was then applied to each article by identifying stressors and potential solutions to crisis while taking into consideration different perspectives. We created a table to represent the compilation of information found.

## Results

**Figure 1:**
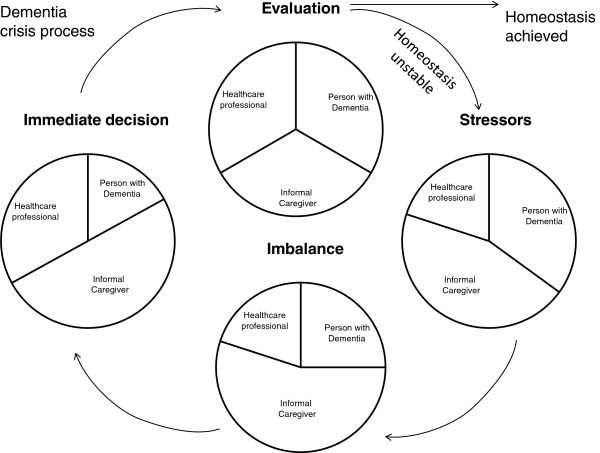
Literature search.

Abstracts and titles of 1,113 articles were screened and narrowed down to 41 articles (see flow diagram in Figure 1). The full text was read by JB and JM-V. Nine of the 41 articles were excluded because they did not mention the word crisis or related terms in connection to dementia care, and five of the 41 articles were excluded because they were not specific to persons with dementia. The remaining 27 articles were included in the review. The publishing dates for the included articles ranged from 1961–2005.

Seven of the 27 articles included a formal definition of crisis (see Table 1). The key features from these definitions included:

• the presence of stressors

• the imbalance created by stressors

• the need for immediate decision

• the view of crisis as a process

• resolution.

**Table 1:** Crisis definitions from seven articles with citations

**Original article**	**Crisis definition**
Hoff, 1995 [7,8]	“The representation of a serious occasion or turning point occurring when an individual is faced with an obstacle that is important to life goals. A crisis is self-limiting because homeostatic mechanisms necessitate resolution of a crisis. A crisis results in depletion of system resources and eventually the system shuts down or ceases to function.”
Caplan, 1961 [7,9]	“An obstacle that is insurmountable through customary methods of problems solving.”
Liken, 2001 [7]	“A process precipitated by a stressor that occurs only in the presence of mediating factors, when normal methods of problem solving have failed, and results in an outcomes or resolution.”
Caplan, 1964 [10,11]	“An imbalance between the difficulty and importance of the problem and resources immediately available to deal with it.”
Butcher and Maudel, 1976 [12-14]	“The dual experience of distress and sense of immediacy associated with a defined, problematic situation.”
Aguilera, 1998 [15]	“A perceived or actual imbalance between perceived difficulty of a life challenge and an available repertoire of coping skills.”
Aguilera and Messick, 1986 [16], Joslin, 1980 [17]	
England, 1994 [17]	“A decision point, an opportunity for growth.”
Maturana and Varela, 1987[18], Shaw and Halliday, 1992 [17]	“In crisis, experience within the niche is detached and out of sync with the rest of the domains of experiences, one or more structural domain.”
Michon, 2005 [19], De Clerq and Dubois, 2001 [20], Aguilera, 1998 [15]	“Periods of disorganization experienced by the entire family that turn into opportunities of change.”

Descriptions of crisis were retrieved and organized into Table 2. The three main perspectives identified in crisis situations in dementia care were:

1) persons with dementia

2) informal caregivers

3) healthcare providers (general practitioners, nurses, and nursing homes).

**Table 2:** Descriptions of crisis from different perspectives

**Operationalized definition of crisis**	**Operationalization**
Filial crisis [12,13]	“Self-reports of distress urgency and inability to engage in usual activities of daily living with a sense of wellbeing as a consequence of caregiving”
Filial crisis in clinical practice [17]	“A condition of urgency, excess emotional arousal, fatigue and difficulty with goal attainment in the caregiving situation”
Evolutionary perspective on filial crisis [17]	“An ongoing period of unfolding of the filial relationship through caregiving”
Implicit operationalization of crisis [21]	“The decision to institutionalize the patient in most cases had been acute when the relatives could not manage the situation anymore”
Crisis experience [17]	“Spontaneously and repeatedly reported episodes of distress and urgency relative to the caregiving situation and inability to engage in usual activities of daily living with a sense of well-being as a consequence of caregiving”
Caregiver crisis [22]	“Where informal skills and commitment are not enough”
Episodic crisis in a nursing home [23]	“Any acute disruptive episode requiring non-routine intervention”
Crisis in nursing home [24]	“Catastrophic psychiatric reactions that are aggressive or attacking”

### Definition of crisis and contents of the operational framework in dementia care

All the dementia-related definitions in the table describe crisis as a critical point in life where stressors should be diminished or homeostasis reinforced to restore balance.

Dementia related crisis is defined as;

a process where there is a stressor(s) that causes an imbalance requiring an immediate decision which leads to a desired outcome and therefore crisis resolution. If the crisis is not resolved, the cycle continues.

**Figure 2:**
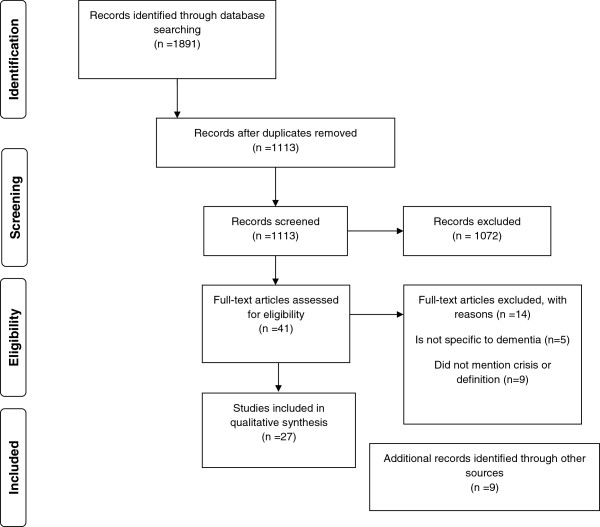
**Operational framework for crisis and retrieving a new equilibrium in dementia care.** The figure represents the full process of crisis in dementia. Dynamic proportions within circles represent the burden and time input for the different perspectives potentially involved. The proportions change to represent the individual situation in a crisis process. Stressor(s) can be psychological, medical, social or environmental change that causes a shift in an individual’s homeostasis. The imbalance represents the resulting state of fragility from the severe breakdown in homeostasis. Immediate decisions aim to regain homeostasis. Resolution equals equilibrium, otherwise the crisis is unresolved.

We propose an operational framework to standardize the definition of crisis based on the perspectives and key features from the seven articles. Figure 2 indicates that there is a stressor, leading to an imbalance in care needs that requires an immediate decision and resolution occurs when there is equilibrium otherwise the crisis cycle continues and no resolution to a homeostasis occurs. The three main perspectives are viewed as interrelated and depend on the type of crisis stressors and where the crisis takes place. The proportion of the involvement of the different perspectives also changes. As dementia severity increases, the role of the informal caregiver and health care provider expands in the desired outcome of the crisis.

In Figure 2, the circle represents the burden and time input for the different perspectives. In the beginning, the person with dementia has the highest amount of stress followed by the caregiver. The health services are minimally involved. However, when a crisis situation occurs, for example, when the caregiver becomes ill, the caregiver may be suddenly overwhelmed by their health problems and the care responsibility for the person with dementia. A decision, usually with consultation from the general practitioner, is made to increase the role of health services. After evaluation of the situation gradually equilibrium is reached and the crisis is resolved.

### Crisis care from the perspective of the person with dementia, the informal carer and the health care provider

This section of the article applies the operational framework to the 27 articles by identifying stressors and potential solutions to crisis while taking into consideration the perspectives of person with dementia, his/her caregiver(s) and the involved health care provider(s) (Table 3). We start with crisis from the perspective of the person with dementia. The next section examines stressors that normally lead to hospitalization or institutionalization with potential solutions involving interactions between the informal caregiver and care in the community. The third section covers stressors and solutions to informal caregiving crisis and lastly, stressors and solutions to crisis in nursing homes.

**Table 3:** Identification of stressors, life imbalance, decisions and crisis outcomes

**Perspective**	**Stressors/predictors of crisis**	**Crisis recommendations**
Person with dementia	Diagnoses [25,26]	1. Counselling [25]
	Inability to live on their own [7,27-31]	1. Lives with family or friends [29,30]
		2. Assisted living [29]
		3. Institutionalization [29,32]
		4. General practitioner assessment [28]
		5. Improved information to caregiver and person with dementia on activities of daily living [27,28,33,34]
		6. Improved information to general practitioner about dementia [27]
	Comorbid conditions [27,28,31,33,35]	1. General practitioner management to detect specific conditions earlier [28,33]
		2. Improved information to health care professionals about dementia [27,33]
		3. Improved information to caregivers [27]
		4. Acute hospitalization/Geriatric home hospitalization [31,33,35]
		5. Structured follow up after hospitalisation [33]
		6. Institutionalization [34]
	Malnutrition [27,28,31,35]	1. General practitioner management [28]
		2. Geriatric home hospitalizations/Hospitalization[31,35]
	Falls [27,28,32,36]	1. General practitioner management [28,33]
		2. Hospitalization [31,35,36]
		3. Institutionalization [32,36]
		4. Improved information to caregiver [27,28,33,34]
		5. Improved information to General practitioner about dementia [27]
		6. Fall prevention program in assisted living facilities [36]
	Behavioural and psychological symptoms of dementia [7,27,28,31,32,37]	1. General practitioner management [22,28],
		2. Careful management of drug therapy [27,31,38]
		3. Improved information to caregiver [28,33,34]
		4. Improved information to General practitioner about dementia [27]
		5. Case management/care consultant [39]
		6. Acute bed assessment of the person with dementia in hospital or psychiatric hospital [37]
		7. Geriatric home hospitalization [35]
		8. Acute hospitalization [31,35]
		9. Structured follow up after hospitalization [33]
		10. Institutionalization [29,32,34]
	Newly institutionalized [10,26]	1. Therapeutic interaction with nurse to promote orientation and psychosocial function [10]
Caregiver	Lack of knowledge [28,31]	1. General practitioner provides information [21,28,31,34]
		2. Carer Support [12,13,17,22]
		3. Care packages [38]
	Miscommunication with general practitioner [34]	1. Clearer communication with the caregiver [34]
		2. Caregiver must be open about caregiving situation [34]
		3. Case management/nurse involvement to assess home situation [34]
	Lack of time for personal or social activities due to increased caring [12,13,17]	1. Temporary respite/temporary admission to nursing home [38,40]
		2. Carer support by community services, professionals, family members [21,38]
		3. Home care [38]
		4. Day care[38]
	Emotional toll of increased dementia severity [26,28,34,41]	1. Introduce care plans [7,17,38]
		2. Carer support by community services, nurse [41], professionals, family members to come up with coping strategies [13,38]
		3. Home care [28,38]
		4. Day care [38]
		5. Temporary respite/temporary admission to nursing home [28,38,40,41]
		6. Institutionalization [34]
	Escalating costs due to dementia severity [38]	1. Customized care plans [38]
		2. Public private partnerships of care offering low cost support services [38]
	Caregiver exhaustion [12,13,19,21,28,29,31,34,40]	1. Advance care planning [7,17,29]
		2. Care plans [17,21,38,39]
		3. Carer support by community services, professionals, family members [13,19,22,31,32,34,38]
		4. Case management [7,13,30,32,38,39]
		5. Social services for patient and caregiver [12,13,31,34]
		6. Home care [38]
		7. General practitioner management of comorbid conditions, caregiving situation, structured follow up after hospitalization [21,28,31,34]
		8. Therapy [13,19,21]
		9. Day care [38]
		10. Temporary respite/temporary admission to nursing home [38,40,42,43]
		11. Hospitalization [31]
		12. Institutionalization [19,21,34]
	Caregiver Illness [13,19,22,28,29,31,34,37]	1. Advance planning [17,29]
		2. General practitioner management [22,28,31]
		3. Community Care Support [22]
		4. Extra day care [38]
		5. Temporary respite/temporary admission to nursing home, hospital or psychiatric hospital [37,38,40]
		6. Acute bed assessment of the person with dementia in hospital or psychiatric hospital [37]
		7. Emergency institutionalization [19,29,34]
		8. Forward planning in cases where the caregiver is old and frail [29]
	Death of caregiver [26,28,29,31]	1. Forward planning in cases where the caregiver is frail [29]
		2. General practitioner management [28,31]
		3. Emergency institutionalization [29]
	Person with dementia institutionalization [7,17,26]	1. Increased preparation for the caregiver [17]
	Death of person with dementia [26]	1. Counselling [26]
Nursing home perspective	Signalling events: physiological, mechanical, psychological, social, or environmental change that affect client status [23,24]	1. Predict and prevent crisis through identification of signalling events [23,24]
		2. Train nursing home staff to identify and appreciate the importance of immediate and gradual changes in behaviour [23,24]
		3. Develop documentation to track signalling events and treatment,
		4. Establish proper procedures for crisis intervention [23,24]
		5. Educate staff to become attuned to subtle changes in the behaviour of persons with dementia who have trouble communicating [23,24]
		6. Identify clients that complain repeatedly as an unmet need may have been overlooked [23,24]
		7. Identify other signalling events and maintain a file for future reference [23,24]
		8. Protection of the person with dementia, other residents and staff [24]
		9. Diversion and environment management [24]
		10. Assessment interventions [23,24]
		11. Increased staff interaction with patients [10,23,24]
		12. Increased family intervention [23,24]
		13. Nurse assistant creates structured program for persons with dementia [24]
		14. Staff should have compassion for persons with dementia [24]

### Crisis from the perspective of the person with dementia

Important stressors leading to crisis from the perspective of the person with dementia are:

• (time to) diagnosis [25,26]

• inability of the person with dementia to live independently [7,27-30]

• unaddressed cormobid conditions [27,31-33,35,37]

• malnutrition [27,28,31,35], falls [27,28,32,36]

• intense behavioural and psychological symptoms [7,27,28,31,32,37]

• being newly institutionalized [7,10,17,26].

Stressors often lead to crises where the outcome was emergency hospitalization or institutionalization.

### Recommendations to prevent crisis after diagnosis disclosure

Diagnosis disclosure of dementia may result in personal crisis where the person with dementia displays “grief reactions related to the experience of actual or anticipated losses associated dementia diagnosis” [25,26]. Designing supportive interventions to maximize adaptive coping responses in the immediate time after the diagnosis is recommended [25,26] (see Table 3).

### Recommendations to address persons with dementia’s inability to live independently

The inability of persons with dementia to live on their own was cited as a stressor leading to a crisis situations like unplanned institutionalization or emergency hospital admission [27-30]. A comparison of emergency and regular nursing home admissions showed that an inability to cope alone was the main reason for referral in both regular and emergency admissions [29]. Solutions included an assessment of living conditions by the general practitioner [28] and different forms of assisted living [29,30,32]. It is unclear how feasible and costly home visits by the general practitioner may be. Improved information to the person with dementia and caregivers on prevention and addressing loss of activities of daily living is recommended [27,28,33,34]. Improved information to the general practitioner about dementia could improve their ability to assist persons with dementia [28,33].

### Addressing comorbid conditions and malnutrition leading to crisis

Earlier prevention, detection and treatment by the general practitioner of acute medical conditions (e.g. syncope and collapse, pneumonia, urinary tract infections, hip fractures and dehydration) and malnutrition is recommended to reduce emergency hospital admissions [28-33]. Natalwala [33] recommends distributing advice to health care providers regarding the most frequent reasons for hospital admission when dementia is involved. Nourhashemi [27] recommended improved information for caregivers with structured follow-up after emergency admission and early management and treatment for the persons with dementia although it is unclear what this entails. Admittance to a nursing home was cited as an option [34].

### Recommendations to prevent falls

Falls are associated with emergency hospitalization and nursing home placement in persons with dementia [27,31-33,35,37]. One study found that persons with dementia who were admitted due to falls and behavioural disturbances were admitted to hospital for the same reasons in the preceding months of the study [27]. Therefore, improved follow up after hospital admission is recommended [27].

Bellantonio [36] investigated whether a multi-disciplinary team intervention compared to usual care led to differences in the time until an “unanticipated transition” for a dementia population living in assisted living. Although an untargeted multidisciplinary intervention (n=48) compared to controls (n=52) did not significantly reduce the risk of transitions for persons with dementia relocating to assisted living, Bellantonio [36] found trends for decreasing hospitalization and death. Falls were the primary reason for nursing home admittance, which lead Bellantonio [36] to recommend a targeted intervention for falls.

### Addressing behavioural and psychological symptoms of dementia

Behavioural and psychological symptoms of dementia were identified as a stressor leading to crisis situations [7,27,28,31,32,37]. Communication between the informal caregiver and the general practitioner was cited as important to general practitioner management [22,28,34]. Several articles recommend improved information about dementia to informal caregivers [28,33,34] and general practitioners [27]. Three articles recommended drug therapy to alleviate some behavioural issues that lead to crisis situations [27,31,38]. Case management is also recommended to alleviate behavioural and psychological symptoms of dementia [39].Several articles recommended an acute bed assessment [37], hospitalization [31,35] and structured follow up [33].

A randomized controlled trial of 109 persons with dementia found that a geriatric home hospitalization service, which provided diagnostic and therapeutic interventions at the home after discharge from the hospital, resulted in a significant reduction in behavioural disturbances and less caregiver stress compared to a hospital ward control group [35]. Geriatric Home Hospitalization with a geriatric team (geriatrician, nurses, physiotherapists, dietician, social worker and counsellor) is open 12 hours a day, seven days a week, plus an out-of-hours emergency plan for caregivers. The authors [35] stated that the positive findings may be due to fewer changes in environment and routine compared to when persons with dementia are hospitalized [35]. Other authors recommended institutionalization when behavioural and psychological symptoms of dementia are too difficult for the informal caregiver to manage [29,32,34].

### Recommendations to approach newly institutionalized persons with dementia

Robinson [10] focused on the crisis of institutionalization for a person with dementia newly admitted to an institution, as they are often disoriented and disorganized in their new environment and have a feeling of loss of control over their lives [10]. Robinson [10] suggested therapeutic interaction between the nurse and the person with dementia through discussing problems, needs and feelings to alleviate the crisis.

### Crisis from the perspective of the informal caregiver

Seven articles identified stressors that lead to crisis from the perspective of the informal caregiver. Stressors included: 

• a lack of knowledge on care for persons with dementia [28,31],

• miscommunication with the general practitioner [34],

• a lack of time for personal and social activities [12,13,17]

• the emotional toll on informal caregivers based on increasing dementia severity [26,28,34,41]

• the escalating costs due to increased dementia severity

• caregiver exhaustion [12,13,19,21,28,29,31,34,40]

• caregiver illness possibly leading to death [7,12,13,17,19,26,29,31,38] institutionalization, [7,17,26]

• death of the person with dementia [26].

### Informal caregivers need for knowledge about dementia

Caregivers require medical and psychological collaborations to help them: 

• overcome difficulties and adapt to their new role associated with dementia care

• prevent caregiver withdrawal, isolation, or burnout

• improve the person with dementia’s general health and well-being [12,13,17,19,21,22,28,31,34].

Care packages tailored to the person with dementia and the informal caregiver could help identify what care is available [38]. Hunter recommends tailored care plans early in the dementia trajectory based on an (unmet) needs assessment for the person with dementia and their carer in addition to highlighting drug therapy, the benefit of carer support through advice and information, support groups, temporary respite in nursing homes, day services and local rehabilitation through community trusts or private public partnerships [38].

### Solutions to miscommunication between the general practitioner and the informal caregiver

A qualitative study identified reasons that delayed appropriate referrals to nursing homes by comparing perspectives of live-in caregivers of persons with dementia (n=21) and the referring general practitioner (n=19) [34]. Reasons for delayed nursing home care included the sense of duty from the caregiver, time constraints leading to inadequate patient assessments and misconceptions on the side of the general practitioner about capability of the caregiver to deal with the person with dementia [34]. The authors advocated the need for clearer communication and better cooperation between caregivers and the general practitioner although it is unclear what this entails [34]. Case management or help from a nurse may also improve communication between the general practitioner and the informal caregiver [7,30,39].

### Time management for informal caregiver’s personal and social activities

Informal caregivers require personal time and the ability to do activities outside of caregiving. Solutions to allow for these activities include; 

• homecare

• carer support from community services (e.g. day care)

• family members and friends [21,38].

Other solutions noted include temporary respite or temporary admission to nursing homes [38,40].

### Solutions for the emotional toll informal caregivers face based on increasing dementia severity

Multiple articles espoused the benefits of a nurse in the home aimed at preventing crises by identifying potential problems and helping caregivers to develop new coping strategies [7,12,13,17,28,30,32,39,41]. England et al. [12,13,17] provide examples where a nurse can be helpful, such as: 

• managing the day to day caring activities

• discharge from hospital planning

• surrogate decision-making in matters of health care

• decisions to institutionalize a family member.

England et al. [17] recommends that concrete goal strategies may lead to greater resolution of interpersonal problems in stressful situations. Additionally, nurses play an important role to assess the caregiving situation by evaluating the caregiver’s interpersonal support networks, their personal emotional stability and their perceived health to predict the caregiver’s ability to sustain their caregiving role [17]. Trained nurses may provide interventions promoting a sense of relatedness with other (informal) carers thereby providing confidence, will and strength for caregivers to continue caregiving [17]. England [12] also looked at crisis support for minorities and recommended expansion of the caregiver’s personal and social support network by telephone and visits, prevention and treatment of caregiver burden with help of professionals, clergy, family and friends.

Case managers are recommended in times of crisis such as hospitalization, acute illness, and problems arising in living arrangements or with their care provider [30]. Other authors advocate case management through periodic visits to the home to assess the caregiving situation and to create a family plan [7,39]. Liken [7] states that health care providers should be attuned to the caregivers’ use of denial to cope with situations and that they should initiate interventions that prevent long-term negative consequences of crisis, like family conflict, sibling resentment, and unresolved guilt. Clark [39] found that physician visits and hospital admissions could be reduced by care consultants (e.g. case managers or trained registered nurses) helping persons with dementia and families initiate timely and regular discussions with general practitioners and other care providers before identified problems escalate into crisis.

General practitioners need to provide basic information to the caregiver on respite care and home support services to avert crisis situations because without such services the crisis may lead to over-burdened caregivers and to unplanned institutionalization or emergency hospital admission [28].

### Escalating costs due to dementia severity

We found one article that cited costs associated with informal caregiver as a stressor for caregiver crisis [38]. Alternative care solutions that could decrease costs for caregivers include partnerships between the public and private sectors to offer low cost support services to delay the need for institutional care [38].

### Solutions to caregiver exhaustion

Balardy [31] concluded that exhaustion of the informal caregiver was an independent and supplementary predictive factor of acute hospitalization.

Preventative measures for caregiver exhaustion included; 

• implement advance care planning [7,17,29]

• create care plans [17,21,38,39]

• involve community services [13,19,22,31,32,34,38]

• participate in day care [38]

• use to case managers [7,13,30,32,38,39]

• involve friends and family members [13,19,22,31,32,34,38].

The general practitioner’s assessment of the caregiving situation could help prevent, detect and treat caregiver exhaustion [21,28,31,34]. Additional treatment for caregiver exhaustion include; therapy [13,19,21], hospitalization [31], temporary respite or temporary admission to nursing home [23,24,38-40] and institutionalization [19,21,34].

### Addressing crisis in times of caregiver illness and death

Crisis admissions to an acute bed unit often occur in instances when the informal caregiver was ill or died [37]. Several authors recommended advanced planning in situations such as when the caregiver is older and frail [7,29]. The following measures are cited as responses to caregiver illness or death:

• general practioner management [22,28,31],

• community care support [22]

• day care [38]

• temporary respite [37,38,40]

• hospitalization [37,38,40]

• emergency institutionalization [29].

### Coping with institutionalization of the person with dementia

Psychological preparation for the caregiver is recommended to deal with the insitituationalization of the person with dementia [22]. Wenger, Scott and Seddon [22] advocated crisis support to change caregiver perceptions of long term care and to involve community care.

### Coping with the death of the person with dementia

One author cited counselling for informal caregivers after the death of the person with dementia [26].

### Crisis from the perspective of the nursing home

A study on nursing homes [23] compared crises with persons with dementia and persons without dementia. Ries [23] defined crisis as “any acute disruptive episode requiring non-routine intervention”. Crises were classified as physiological (temperature), mechanical (falls), psychological (distress) or combined. In both groups, physiological crises had the highest prevalence followed by mechanical crises (e.g. falls) [23].

Paulmeno [24] described crisis in nursing homes as “catastrophic psychiatric reactions” from persons with dementia. Violent crisis scenarios (e.g. attacking someone) were often accompanied by friction between care providers and family members. The friction was in regard to the opted crisis management [24].

### Solutions to nursing home crisis

Two articles found that crisis situations could be avoided in nursing homes if staff became more attuned to signs and symptoms of distress in persons with dementia [23,24].

Recommendations to prevent crisis in persons with dementia include a therapeutic environment with nursing staff who can provide a structured programs and who are able to predict and prevent crisis through identification of signalling events [23,24]. Recommendations also included training nursing home staff to: 

• identify and appreciate the importance of immediate and gradual changes in behaviour

• notice subtle changes in the behaviour of persons with dementia who have trouble communicating

• notice an any unmet needs that may have been overlooked

• identify other signalling events

• keep a file for future reference

• develop documentation to track signalling events and treatment

• establish of proper procedures for crisis intervention [23,24].

Better and non-confrontational communication with informal caregivers is also advised, although it is unclear if that meant more meetings or one specific quality of communication [23,24].

## Discussion

This is the first article to review the definition of crisis in the context of dementia care. We think it is important to have a standardized definition of crisis for use in clinical practice, health policy and research in order to properly communicate and find constructive solutions. The focus of this review is on the compilation of perspectives, stressors and recommendations in dementia crises. We were as inclusive as possible in order to understand how the word crisis is used in the literature. We could not find any literature pertaining to crisis in a hospital environment. This is concerning because we believe hospitals are not always conducive to dealing with the idiosyncrasies of persons with dementia (e.g. hostility and wandering). Nourashemi [27] recommended increased consideration to the organization of emergency facilities to enable an adequate response to the medical problems raised by persons with dementia.

The current review of the literature on crises with persons with dementia synthesized the information of 27 articles and generated a more standardized definition of crisis. We defined dementia related crisis as *a process where there is a stressor(s) that causes an imbalance requiring an immediate decision which leads to a desired outcome and therefore crisis resolution. If the crisis is not resolved, the cycle continues.* Persons with dementia, caregivers and health support staff create a delicately balanced circle that enables a new equilibrium for different phases in the continuum of dementia care. During the dementia trajectory, there are certain times when a serious disruption in the care situation creates a need for an immediate decision. There is no resolution without a change that results in a new equilibrium.

The person with dementia, the caregiver, and the health support staff all have different perspectives on the definition of crisis and its resolution. For the person with dementia, change and loss of control is a very personal challenge that causes great anxiety and frustration. The person with dementia may be unable or unwilling to accept the change and retreat into more severe symptoms or depression. The caregiver may be unable to cope with certain reactions from their loved one(s) during the crisis and therefore feel tremendous guilt. The health care support workers may look at the crisis situation of the caregiver and the person with dementia and be unable to empathize with an untenable situation. From the literature, it becomes clear that crises come in all kinds of formats and that every crisis has a unique solution. Therefore, it is very difficult to prevent or plan for all crises even if there is a willingness to create a strategic plan.

Having defined dementia crisis, we created a framework of crisis in dementia care. We used this framework to compile crisis stressors and recommendations from the literature based on different perspectives. Recommendations for persons with dementia in the community include awareness therapy after diagnosis, increased contact with the general practitioner to control comorbid conditions, case manager consultations, caregiver support and education. Nursing home staff needs to be attuned to the environmental, physical and psychological needs of persons with dementia. Additionally, constructive communication with the family was mentioned.

We recommend future research use the operational framework for crisis in a dementia care setting, for example by evaluating records based on dementia crisis support. This could also help identify if there are stressors associated with hospitalization, hospital preparedness, patient support and caregiver guidance. After its validation in the various care settings, we recommend that the framework be used in the planning of care services and research. The framework may also have the potential to attach costs to crisis situations. This would allow for a monetary estimation of avoiding or handling crisis situations in dementia care.

## Conclusions

This is the first article to review the definition of crisis in the context of dementia care. A review of the literature indicated that the definition of a crisis is idiosyncratic. Therefore, it is difficult to prevent or plan for all crises. We used an operational framework to compile types of crisis stressors and recommendations from the crisis literature based on three different perspectives; the person with the dementia, the caregiver and the healthcare providers.

## Competing interests

The authors declare that they have no competing interests.

## Authors’ contributions

JMV and JEB carried out the literature review. JMV, JEB, and SER were involved in the design, analysis and interpretation of data. All authors helped draft the manuscript. All authors read and approved the final manuscript.

## Pre-publication history

The pre-publication history for this paper can be accessed here:

http://www.biomedcentral.com/1471-2318/13/10/prepub
